# Medical News

**Published:** 1852-01

**Authors:** 


### MEDICAL NEWS.

University of the City of New York.—The medical department of this university have lately announced some changes in their system of instruction. The winter course of lectures is to be conducted, as heretofore, by the six professors constituting the Medical Faculty. On the 15th of March, a second course of lectures begins, which is to be continued until the following October. Lectures during this summer term are to be given, in part, by the professors who lecture during the winter; but in addition, a new corps of professors has been appointed, whose lectures are to be given solely during the summer. At the head of the list of the latter is the name of Prof. Charles A. Lee, who is to lecture on medical jurisprudence. The other professors are as follows: B. W. Macready, on hygiene and toxicology; Thomas M. Markoe, pathological and microscopic anatomy; W. H. Van Buren, diseases of the genito-urinary organs; J. T. Metcalf, physical diagnosis and diseases of the chest; Dr. Bulkley is to give a course of lectures on diseases of the skin.

The faculty, in their announcement, state that this new plan has been adopted in order to enlarge the time and opportunities for medical instruction, and under the belief that students cannot be *coerced* to pursue a more extended course than that required by medical colleges generally. The advantages of the summer course are optional with the student, but it is proposed to confer on those who avail themselves of these advantages, certificates of honor.

We have not time, nor space, to comment on the above plan at this time. We will only remark that the university has been fortunate in having been able to secure the services of Prof. Lee. We may be allowed to say this, without intending any invidious reflections on the gentlemen associated with him.—*Buffalo Med. Journal.*

Veterinary Journal.—A *veterinary journal* is issued at Boston, under the editorial charge of George H. Dodd, M. D.

Primary Medical School.—At an early period after its organization, the American Medical Association recommended the establishing of Primary Medical Schools, and in accordance with this recommendation Dr. F. R. Ramsey, of Knoxville, Tenn., announces the intention of opening such a school.

Dr. Ramsey will endeavour to direct the attention of his pupils to such books as will convey most comprehensively, and in the order here mentioned, a knowledge of Anatomy, Physiology, Pathology, Materia Medica, Therapeutics, Practice of Medicine, Obstetrics, and Surgery.

Examinations, and oral explanations, with illustrations by paintings, preparations, and apparatus, will constitute very material elements in the contemplated course of instruction.

The class will be formed on the 15th day of February, 1852.

The terms for the entire course of nine months, will be fifty dollars.

Should the size of the class render it necessary, the assistance of physicians amply qualified to instruct will be obtained.

Dr. Ramsey is a gentleman of great zeal and undoubted talents, and we commend his project to the attention of southern students.—*Medical News.*

Cost of the Doctorate in Paris.—The Union Médicale makes the following estimate of the cost of the Degree of Doctor of Medicine in Paris: The collegiate education requires 7 years, and to obtain the two baccalaureate degrees, 2 years more are necessary; then the medical studies, properly speaking, will average 6 years; making a total of 15 years. The 7 years at college cost 1000 francs per annum, making 7000 francs; the 2 baccalaureates, 320 francs; the 6 years at medical college, 1200 francs a year, or total 7200 francs. Private courses of study, 1000 francs; matriculations, examinations, and diploma fee,

1100 francs; instruments and books, 2000 francs. Making a grand total of 18,620 francs, or about $3,724.

Action of Water on Lead Pipes.—The attention of readers is requested to the following circular of the Committee appointed at the last meeting of the American Medical Association. It is only by a generous and hearty assistance from their professional brethren that the full benefit of these committees is to be obtained.

To the Members of the Medical Profession in the United States.—The undersigned, a Committee of the American Medical Association to report on "the action of water on lead pipes, and the diseases which proceed from it," are desirous of obtaining from their professional brethren any information that is calculated to throw light on this important, but hitherto generally unobserved subject. They therefore take the liberty of proposing the following questions.

1st. Have you, in your practice, met with cases of lead or painter's colic produced by using water drawn through lead pipes, or contained in leaden cisterns?

2d. Have you met with cases of arthrolgy? If so, have they been attributable to this cause?

3d. Have painful neuralgic diseases been observed by you, among persons using water thus exposed to lead?

4th. Have you seen instances of lead encephalopathy?

5. Have you observed paralysis as a precursor, concomitant or sequel to either of the above forms of disease?

Answers to any or all the foregoing questions, and any facts or information as to any form of disease originating in the use of water impregnated with lead, will be very gratefully received. Accurate descriptions of all cases would be desirable, especially their early history. It will also be very important to know the length of time each individual case had been exposed to lead before the disease became manifest.

As the report must be made at the annual meeting of the Association to be held in Richmond, Va., in May next, it is desirable that all information should be forwarded to any one of the Committee previous to the first of March next.

Horatio Adams, Waltham, Mass.

Sam'l L. Dana, Lowell, "

John C. Dalton, " "

} *Committee.*

*Waltham, December 5th,* 1852.—*Boston Med. and Surg. Journal.*

COMMITTEE ON THE RADICAL CURE OF REDUCIBLE HERNIA.—*To the Members of the Medical Profession throughout the United States:* The undersigned are a Committee of the American Medical Association, to report on "the radical cure of reducible hernia." They are desirous of obtaining from their professional brethren any information that is calculated to throw light on this important and interesting subject.

They therefore take the liberty of proposing the following questions. An answer to any or all of them, or any facts connected with the branch of surgery on which they are directed to report, would be gratefully received:

1st. Have you been in the practice of treating reducible hernia with a view to its radical cure?

2d. Have you ever performed any surgical operation for this purpose?

3d. If so, please to describe the operation, and the mode of performing it.

4th. What proportion of cases, of all in which you have operated, has been cured?

5th. Have any alarming or fatal effects, in any instance, been caused by the operation?

6th. If so, please to describe them.

As the Report must be made at the Annual Meeting of the Association, to be held in Richmond, Va., in May next, it is desirable that the answers to the above questions shonld be forwarded to any one of the Committee on or before March 1st, 1852.

GEO. HAYWARD,<br>J. MASON WARREN,<br>S. PARKMAN, } *Committee.*

*Boston, November 26th,* 1851. *N. H. Journ. Med.*

"EPIDEMIC JAUNDICE" has been for three weeks past and is at present very prevalent in Selma, Alabama. We have in this pleasant little town, situated on the Alabama river 300 miles above Mobile, a population of about fifteen hundred inhabitants, white and black, young and old, (a New England village,) and we are able to number *fifteen regular practitioners of medicine!*

GEORGE ORD, Esq., has been chosen President of the Academy of Natural Sciences of Philadelphia, to succeed the late Dr. S. G. Morton.

DEATH OF DR. R. L. HOOPER.—It is with much regret that we have to record the death of Dr. Robert Little Hooper, who died on Friday the 7th inst., from concussion of the brain, consequent upon being thrown from his carriage, the horses having taken fright. Mr. Hooper commenced general practice about twenty-seven years ago, in the London road, and for many years had one of the most lucrative and extensive practices on the Surrey side of the water. He was for a long period assistant-surgeon and surgeon to the Queen's Prison; surgeon to the parish of St. George the Martyr, Southwark, to the National Guardian Institution, Bedford row, and to the Yorkshire School. Mr. Hooper, some time last year, took his degree of M. D. at St. Andrew's, or at Aberdeen, and, we believe, had retired from the more laborious duties of his profession, having lately taken an active partner. Dr. Hooper was well known to the Fellows of the Medical Society of London, at which he was a frequent attendant, and had filled several posts of honor in connexion with it. Dr. Hooper, for many years past, had been an active medical reformer, was one of the Council of the British Medical Association, and belonged to the Committee of Poor law Officers. He was an unflinching advocate of the rights of the profession, and indefatigable in his efforts to redress its wrongs. He will be deeply lamented by his bereaved family, and by a numerous circle of professional friends and patients, to whom he was endeared by kindness, urbanity, and integrity.—*Lancet.*

Testimonial to Dr. Lever.—A very pleasing tribute of respect and friendship, as well as acknowledgment of professional skill, has just been received by the family of J. C. W. Lever, Esq., M. D., (one of the Physicians of Guy's Hospital,) from a number of Ladies, being patients of his. It consists of a marble bust of the doctor, executed in the first style of the art, by Baily, R. A. The presentation of the bust was made, on behalf of the subscribers, by their treasurer, Mr. Joshua W. Butterworth.—*Ib.*

Deaths from Chloroform.—The Medical Gazette of Strasbourg furnishes the particulars of a case in which a lady suddenly died during the extraction of a tooth under the influence of chloroform. Another death occurred last July, in London, during an operation for the removal of the testis under the influence of this agent.

A female, thirty-seven years of age, named Elizabeth Hollis, the wife of a laborer in Chipping Norton, England, died lately from the effects of chloroform, administered prior to an operation by Mr. John Farwell, surgeon of that place. At the inquest it appeared that the deceased had long suffered severely from a cancer of the uterus, and that an operation of some kind, nature not stated, being contemplated, chloroform was given, with a fatal result. The mode of giving the anæsthetic was not mentioned at the inquest, or, at all events, is not stated in the newspaper report, which is very meagre. The jury were perfectly satisfied that the chloroform was necessarily used, and properly and judiciously applied under the circumstances, and returned a verdict accordingly. Mr. Farwell should furnish the profession, for their satisfaction, with all the circumstances of the case, the symptoms and progress of the disease, the nature of the operation he proposed, the mode in which the anæsthetic was given, the fatal symptoms it produced, and the treatment adopted to combat them. We recommend Mr. Stanley's lecture, recently published in this journal, to his consideration, and that of all surgeons. It is eminently practical.—*Medical Times.*

